# Evaluating the Evidence: Is Neurolysis or Neurectomy a Better Treatment for Occipital Neuralgia?

**DOI:** 10.7759/cureus.11461

**Published:** 2020-11-12

**Authors:** Sarah McNutt, David R Hallan, Elias Rizk

**Affiliations:** 1 Neurosurgery, Penn State Health Milton S. Hershey Medical Center, Hershey, USA

**Keywords:** neurolysis, neurectomy, occipital neuralgia, surgery, headache

## Abstract

Occipital neuralgia, a neuropathy of the occipital nerves, can cause significant pain and distress, resulting in a decrease in the patient’s quality of life. Options for surgical treatment involve transection or decompression of the greater and lesser occipital nerves. Current evidence provides no clear consensus regarding one technique over the other. Here, we present a systematic review of the literature to potentially answer this question. Eligible studies compared neurolysis versus neurectomy for the treatment of occipital neuralgia after failure of conservative therapy. Our outcome of interest was resolution of symptoms. We performed a search of MEDLINE/PubMed and Ovid from inception to 2019. Eligible studies included the words "occipital neuralgia" and "surgery." All studies comparing neurolysis to neurectomy were included in the analysis. None of the studies identified were randomized control trials. Each study was evaluated by two independent researchers who assigned a level of evidence according to the American Association of Neurology (AAN) algorithm. Data extracted included mechanism of surgery (neurolysis or neurectomy), resolution of pain symptoms, and length of follow-up. Each study was level IV evidence. After reviewing the data, there was insufficient evidence to recommend one method of treatment over the other. This inconclusive result highlights the importance of a national registry to compare outcomes between the two treatment modalities.

## Introduction and background

Occipital neuralgia (ON) is a neuropathy of the greater and/or lesser occipital nerves. The occipital nerves originate in the dorsal ramus of C2, deep to the inferior oblique [1]. The medial branch, the greater occipital nerve, follows the inferior oblique muscle, covered by the splenius capitis and longissimus, and eventually penetrates the semispinalis capitis muscle [1]. Subsequently, the nerve enters the aponeurotic fibrous attachment of the trapezius and sternocleidomastoid, following the superior nuchal line, where it eventually innervates the posterior scalp [1]. The lesser occipital nerve ascends along the sternocleidomastoid, penetrating the fascia near the cranium to continue toward the occiput, where it innervates the posterior auricular surface and bordering posterior scalp [1].

ON often occurs as a result of compression of the greater occipital nerve as it courses through the trapezius muscle aponeurosis [2]. Idiopathic causes of greater or lesser occipital nerve compression include post-traumatic tissue scarring, mechanical damage, irritation, or vascular compression [2,3]. Artero-venous dural fistula, rheumatoid arthritis, whiplash injury, vertebral venous plexus engorgement, tumor, infection, or herpes zoster are all less frequent etiologies of ON but must be considered [1,2-5].

Approximately 0.7% to 13% of pain in headache patients is due to ON [1]. Criteria for diagnosis include sudden, stabbing pain in the dermatomal distribution of the greater or lesser occipital nerve with or without aching between attacks, sensitivity on palpation, and alleviation with local anesthetic [2-7]. Corticosteroids or local anesthetic injections can be both helpful in diagnosis and in providing temporary pain relief [1,3]. In most cases of ON, conservative treatment with nonsteroidal anti-inflammatory medications and acetaminophen is generally effective but often only provides temporary relief [1]. Narcotics play a minimal role in the treatment and are often ineffective [1].

On occasion, injections of local anesthetic with or without concurrent corticosteroids have provided permanent relief of symptoms [4]. Botox injections have demonstrated efficacy, but the period of pain relief is often limited [4,6]. When pharmacology, injections, and other conservative measures fail, other options include radiofrequency ablation or nerve stimulator implantation [1,3,7]. In refractory cases, more aggressive surgical treatment such as rhizotomy, nuchal muscle release, or radiofrequency thermocoagulation of the nerve may be indicated [3,5,7,8]. However, these procedures have a high incidence of treatment failure, including neuroma formation, and are now rarely used [3,8]. Therefore, neurectomy (NR) and neurolysis (NL) are more frequently performed [3].

A limited number of studies directly comparing NL and NR exist in the literature, all of which are >10 years old. Here, we provide an up-to-date literature review of NL and NR for treatment of ON with the goal of providing clarity regarding differences between the two approaches and a recommendation on the superiority of one treatment over the other.

## Review

Study selection criteria

**Figure 1:**
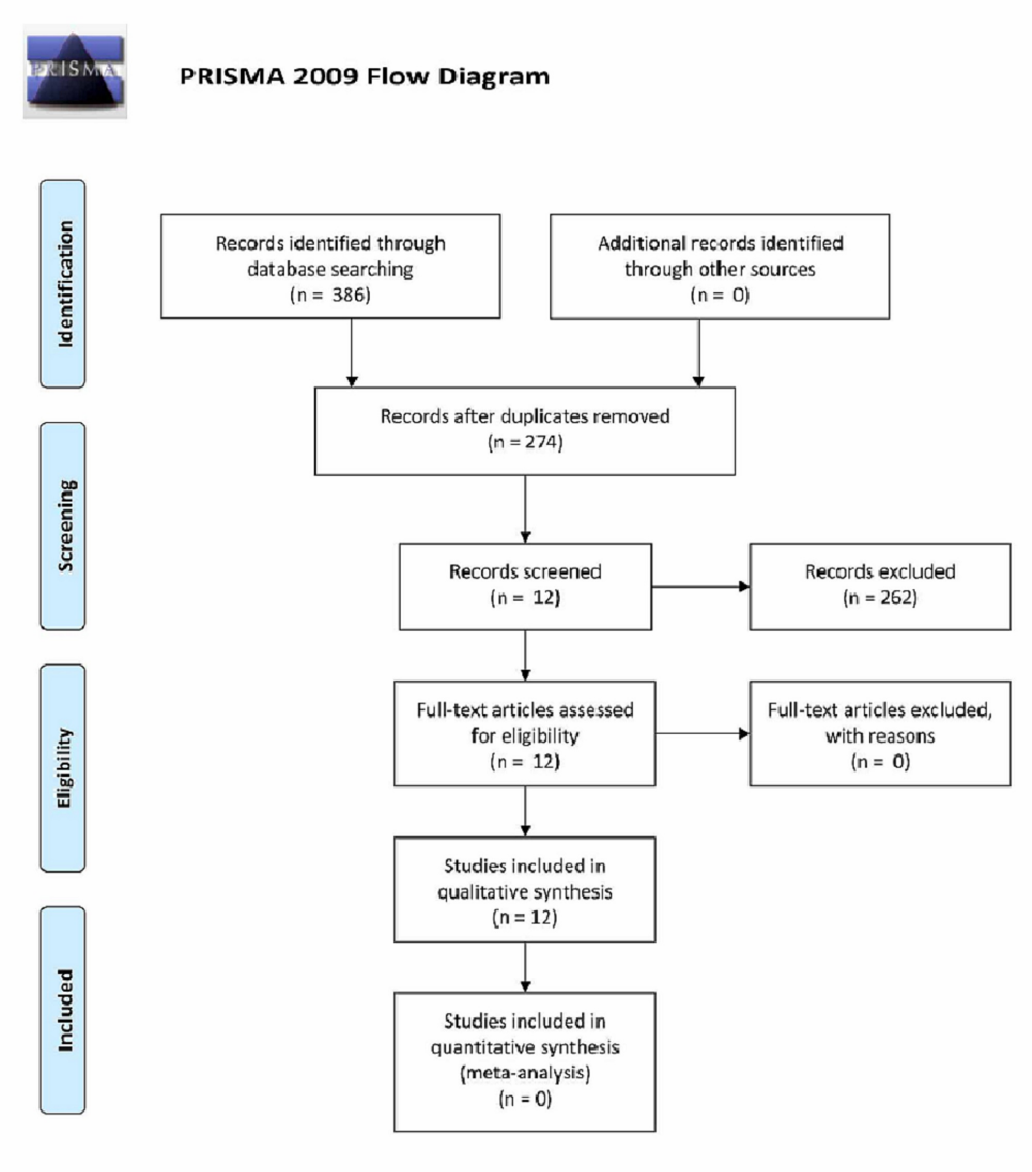
PRISMA Flow Diagram

Studies utilized for our analysis included those that compared NL versus NR for the treatment of ON after conservative therapy failure. A computerized search of MEDLINE/PubMed and Ovid from inception to 2019 was performed using the keywords "occipital neuralgia" and "surgery." Age was not an exclusion criterion as all patient ages were included. However, only articles in English were included. Additionally, similar to other published articles on this topic, articles were restricted to those published within the last 10 years. The preferred reporting items for systematic reviews and meta-analyses (PRISMA) guidelines were implemented (Figure 1).

No randomized controlled trials were identified in the literature.

Data synthesis and analysis

Each study included in our analysis was assigned a level of evidence according to the American Association of Neurology (AAN) algorithm by two reviewers (Table 1). There were no disagreements between the two reviewers on an article's level of evidence. Every study included was determined to be class IV; thus, a meta-analysis was not performed.

**Table 1:** Level of evidence according to the American Association of Neurology algorithm. Table reproduction approved by Payne et al. [9]. AAN: American Association of Neurology.

AAN level of evidence	AAN description
Class I	A cohort with prospective data collection. All relevant confounding characteristics are presented and substantially equivalent between comparison groups, or there is an appropriate statistical adjustment for differences. Outcome measurement is objective or determined without knowledge of risk factor status. Primary outcome(s) are defined, exclusion/inclusion criteria are defined, and dropouts are accounted for (dropout rate is less than 20%).
Class II	Cohort study with retrospective data collection or case-control study. All relevant confounding characteristics are presented and substantially equivalent among comparison groups, or there is an appropriate statistical adjustment for differences. There is masked or objective outcome assessment. Primary outcome(s) are defined, exclusion/inclusion criteria are defined, and dropouts are accounted for (dropout rate is less than 20%).
Class III	Cohort or case-control study. There is a description of major confounding differences between risk groups that could affect outcome. Outcome assessment is masked, objective, or performed by someone other than the investigator who measured the risk factor.
Class IV	Study did not include persons at risk for disease. Study did not include patients with and without the risk factor. There is an undefined or unaccepted measure of risk factor or outcome. No measure of association or statistical precision is presented or calculable.

Additionally, study information including the number of patients, surgery performed, outcome, and mean follow-up time was recorded. In accordance with other works, we used the grading recommendations, assessment, development, and evaluations (GRADE) system to assess the level of evidence of the available literature [8,9]. The GRADE guidelines (Table 2) were utilized to designate the available body of evidence as very low. The literature reviewed in this study mainly consisted of observational studies, making our initial designation low. However, due to the number of case reports in our body of literature, in addition to a consistent lack of comparison group in many studies, we modified our designation to very low. Additional reasons for downgrading our designation include high dropout rates, low population number, discrepancies between treatment group sizes, lack of study blinding, and lack of correlating outcomes to a particular surgical treatment.

**Table 2:** GRADE guidelines. Table reproduction approved by Payne et al. [9].

Study design	Initial quality of evidence	Factors that decrease the quality level	Factors that increase the quality level
Randomized trials or double-upgraded observational studies	High	High likelihood of bias	Large effect
Downgraded randomized trials or upgraded observational studies	Moderate	Indirectness of evidence	All plausible confounding would reduce a demonstrated effect or suggest a spurious effect if no effect was observed
Double-downgraded randomized trials or observational studies	Low	Imprecision	Dose response gradient
Triple-downgraded randomized trials, downgraded observational studies, or case series/reports	Very low	High probability of publication bias	N/A

Results

Our initial search returned 348 articles, 74 of which were duplicates. After screening titles and abstracts, we found 12 articles that directly addressed the question of NL vs NR for the treatment of ON (Table 3) [1-12].

**Table 3:** Neurectomy vs Neurolysis. AAN: American Association of Neurology.

Author	Published in	Study type	Neurectomy or neurolysis	Number of patients	Outcome	Mean follow-up	AAN level of evidence	GRADE criteria
Andrychowski et al. [2]	Folia Neuropathologica	Case Report	Neurolysis then neurectomy x2	1	Relapse of symptoms within one month after neurolysis. No resolution after first neurectomy. Elimination of pain after second neurectomy.	6 months	4	Very low
Ducic et al. [1]	Plastic and Reconstructive Surgery	Retrospective Review	Neurolysis	190	166 (80%) had greater than 50% relief of pain. 72 (43.4%) had complete relief. 40 (19.5%) had less than 50% relief of symptoms.	12 months	4	Low
			Neurectomy	16				
Cornely et al. [7]	Headache	Case Report	Neurolysis	1	Neuralgic pain remained absent.	12 months	4	Very low
Jung et al. [5]	Korean Journal of Pain	Case Report	Neurectomy	1	Headache disappeared gradually, despite the fact that he had discontinued all pain medications.	5 months	4	Very low
Li et al. [10]	Turkish Neurosurgery	Prospective Review	Neurolysis	76	Headache symptoms of 68 (89.5%) completely resolved; another five (6.6%) patients were significantly relieved without the need for any further medical treatments. Three (3.9%) experienced recurrence. All experienced hypoesthesia of the innervated area and recovered gradually within one to six months.	20 months	4	Low
Pisapia et al. [3]	World Neurosurgery	Retrospective Review	Neurolysis	11	19 (66%) experienced a good or excellent outcome with no difference in mean pain reduction among the three cohorts.	5.6 years	4	Low
			Neurectomy	10				
			Neurolysis + Neurectomy	8				
Ducic et al. [11]	American Headache Society	Retrospective Review	Neurectomy	7	Six experienced pain reduction and improvement in quality of life of greater than 80%.	32 months	4	Low
Ducic et al. [12]	Annals of Plastic Surgery	Retrospective Review	Neurectomy	71	41% of patients showed a 90% or greater decrease in symptoms. Bothersome numbness or hypersensitivity in the denervated area in 31%.	33 months	4	Low
Choi et al. [4]	Acta Neurochirurgica	Retrospective Review	Neurolysis	68	47 (69.1%) achieved excellent or good results.	5 years	4	Low
Ko et al. [13]	Journal of Neurological Surgery	Case Report	Neurolysis	1	Resolution.	12 months	4	Very low
Jose et al. [6]	Journal of Craniofacial Surgery	Prospective Review	Neurolysis	11	Three reported complete elimination of pain, six reported significant relief. Two failed to notice any improvement.	12.5 months	4	Low
Janjua et al. [14]	Journal of Clinical Neuroscience	Case Report	Neurectomy	1	Pain free.	2 months	4	Very low

All articles were evaluated by two independent reviewers and assigned a class of evidence. Figure 1 demonstrates a PRISMA flow diagram of our study selection protocol.

Discussion

The low quality of evidence made a meta-analysis not possible. The 12 studies identified utilized varying inclusion and exclusion criteria in addition to outcome measures. Some studies excluded iatrogenic ON; others did not. Some studies included patients with prior surgical management, whereas some studies excluded them. Many studies did not comment on postoperative numbness or surgical complications. The outcome measure for some studies was symptom resolution, and for others, symptom relief. All studies were observational or case reports. Only one study, by Pisapia et al. directly compared neurectomy versus neurolysis for resolution of pain and found no statistical difference in symptom resolution [3].

In 2009, Andrychowski et al. presented a case report on a patient that failed conservative management with pharmacology and 2% xylocaine nerve blocks and subsequently underwent neurolysis of the greater occipital nerve and the occipital artery using Tachocomb pledgets [2]. After one month, the patient failed to have resolution of symptoms and subsequently underwent two neurectomies. The second neurectomy transected the nerve more proximally after the initial surgery failed to resolve symptoms. The patient experienced elimination of pain at six-month follow-up.

Ducic et al. performed a retrospective review in 2009 [1]. One hundred and ninety patients underwent neurolysis of the greater occipital nerve, 12 (6%) underwent greater and lesser occipital nerve neurectomy, and four (2%) had lesser occipital nerve neurectomy. The greater occipital nerve was dissected both proximally and inferiorly to release the nerve from the semispinalis capitis and obliquus capitis, respectively. Furthermore, the nerve was dissected distally to release the nerve from the site of penetration through the trapezial fascial attachment to the occiput. With a mean follow-up of 12 months, 166 patients (80%) had greater than 50% relief of pain, whereas 40 patients (19.5%) had less than 50% relief of pain. Of the 166 patients that had greater than 50% relief of pain, 72 of them (43% of the total number of patients) experienced complete resolution of symptoms. Pain was assessed using the 0 to 10 visual analog scale as well as the migraine headache index [days/months x intensity (0-10) x duration (fraction of 24 hours)] [1]. A direct comparison of neurolysis versus neurectomy and the effect on patient-specific outcomes was not performed in this paper. 

In 2010, Cornely et al. presented a case report of greater ON that failed conservative management due to a vascular loop of the occipital artery [7]. Their patient had complete resolution of symptoms, as measured by the visual analog scale, at 12 months of follow-up after undergoing a neurolysis. 

In 2011, Jung et al. presented a case report of a patient that, after undergoing a C1-3 fusion due to trauma, developed bilateral stabbing pain in the distribution of the greater and lesser occipital nerves [5]. Permanent pain relief could not be achieved through pharmacology, nerve blocks, or radiofrequency thermocoagulation. Subsequently, the patient underwent neurectomy using transcranial doppler sonography to locate the occipital artery and thus the greater and lesser occipital nerves. At five months the patient was experiencing a gradual resolution of symptoms. 

Li et al. performed a prospective review in 2011 [10]. Seventy six patients underwent neurolysis. At an average of 20 months follow-up, headache symptoms completely resolved in 68 patients (89.5%), and five patients (6.6%) were significantly relieved without the need for any further medical treatments. However, three patients (3.9%) experienced a recurrence of their symptoms. All 68 patients experienced hypoesthesia of the area innervated by the lysed nerves, which recovered gradually within one to six months. 

Pisapia et al. performed a retrospective review of 29 patients in 2012 [3]. Eleven patients underwent neurolysis, 10 patients underwent neurectomy, and eight patients had both operations. The patients themselves chose which procedure they wished to undergo. The decompression was performed by identifying the C2 nerve root and ganglion and dissecting free the ligament, adjacent scar, and venous elements, with inferior hemilaminotomies as needed. Neurolysis was performed proximal and distal to the C2 ganglion, removing the ganglion en bloc. Outcome measures were visual analog scale postoperatively compared to preoperatively, patient satisfaction, disability, and quality of life. Nineteen patients (66%) experienced a good or excellent outcome with no difference in mean pain reduction or outcome rating among the three cohorts at an average of 5.6 years of follow-up. 

In 2012, Ducic et al. performed a retrospective review of seven patients who underwent neurectomy for ON occurring after acoustic neuroma resection via retrosigmoid approach [11]. Outcomes measured included migraine headache index, number of pain medications used, patient satisfaction, and change in the quality of life. With a mean follow-up time of 32 months, six patients (85%) experienced pain reduction and improvement in quality of life of greater than 80% and reported being “very satisfied” with the results of the procedure. The use of pain and headache medications decreased on average from 6.4 to 2.4 medications per patient postoperatively.

In 2014, Ducic et al. performed a retrospective review of 71 patients who underwent neurectomy after failed maximal medical management and previously underwent occipital nerve decompression with a mean follow-up of 33 months [12]. Migraine headache index, migraine disability assessment questionnaire, surgery satisfaction, and quality of life were the outcomes measured. Postoperatively, the average reduction in migraine headache index was 63%. Fifty patients (70%) achieved a reduction of 50% or more using the migraine headache index. The average pain level preoperatively was 7.59 and 4.55 postoperatively, for an average reduction of 3.04 (40%). The average migraine disability assessment score that measures the quality of life and days missed from work or social functions was 169.4 preoperatively and 81.89 postoperatively, for an average reduction of 87.55 (49%). Forty eight patients (74%) were happy with their greater occipital nerve excision surgery. Forty-one percent of patients showed a 90% or greater decrease in symptoms. 

Choi et al. performed a retrospective review in 2015 of 68 patients who underwent neurolysis [4]. The C2 nerve root and ganglion were proximally and distally decompressed via excision of adjacent scar, ligament, and vascular elements. Outcome measures were determined via the visual analog scale and medication use. The mean visual analog scale preoperatively was 7.5 and 2.1 postoperatively. Forty seven patients (69.1%) achieved excellent or good results at a mean of five-year follow-up.

In 2017, Ko et al. reported a case of hemifacial trigeminal pain referred from ON due to compression of the greater occipital nerve by the occipital artery [13]. The occipital artery was superficial to the greater occipital nerve, creating a crossing point and a constricting vascular loop. The patient underwent neurolysis and enjoyed complete resolution of pain at 12-month follow-up.

Jose et al. performed a prospective review of 11 patients that underwent neurolysis of the greater occipital nerve for their ON [6]. A piece of the semispinalis capitis muscle adjacent to the greater occipital nerve was removed, allowing distal dissection to release the nerve from the trapezial fascia. The occipital artery was dissected and ligated if impinging the nerve. Three patients (27%) reported complete elimination of pain, six (54%) reported significant relief, and two (18%) failed to notice any improvement at an average of 12.45 months of follow-up.

Janjua et al. reported a case of ON that responded to neurectomy at two-month follow-up [14]. The C2 nerve root, ganglion, and rootlets were identified and dissected proximally. The nerve proximal to the C2 ganglion was cauterized, circumferentially ligated with 4-0 neurolon, then cut with a surgical blade. The C2 nerve and ganglia are excised en bloc.

In our literature review, multiple cases report favorable outcomes in up to 89.5% of patients who underwent neurolysis [1,3,6,7,10,11]. Others such as Ducic et al. had unfavorable results, resulting in salvage neurectomies [2,8,13]. There is no consistent outcome in the literature for neurectomies. Some report success in up to 85% of their treatment group, while others have only experienced unfavorable results [5,13]. Therefore, we are unable to comment on which treatment is superior.

It is important to remember that many patients experiencing ON have concomitant headache diagnoses, and the treatment of the ON may not relieve all of their symptoms [5,6,11]. Additional confabulators such as depression, other medical conditions, and life stressors were not addressed in these studies. Aggressive screening for other causes of occipital headaches must occur; otherwise failure of treatment may occur [13]. Additionally, it is important to remember that failure of surgical treatment of ON could be due to anatomical variation of the greater occipital nerve stem and the location of its divisions into branches [2].

Factors correlated with a positive outcome after surgery, whether neurectomy or neurolysis, included a positive response to greater occipital nerve block or Botox, tenderness over the greater occipital nerve, and being under the care of a specialized neurologist or pain specialist [11]. Longer duration of headache (greater than 13 years) and retro-orbital/frontal radiation have been correlated with therapeutic failure [7].

## Conclusions

A review of the literature comparing NR to NL revealed no studies favored or recommended one modality of treatment over the other. As a result, we are unable to comment on the superiority of one treatment over the other. Both NR and NL are widely accepted surgeries for symptom relief in treatment-resistant ON. However, without higher-quality studies, we will continue to be unable to comment on which treatment is superior. Ideally, a randomized control trial would be implemented to evaluate these surgical options. Alternatively, the creation of a prospective national registry would assist in answering this clinical question as well as other questions within this population.
